# Structural and Optical Properties of High Entropy (La,Lu,Y,Gd,Ce)AlO_3_ Perovskite Thin Films

**DOI:** 10.1002/advs.202202671

**Published:** 2022-08-26

**Authors:** Zachary J. Corey, Ping Lu, Guangran Zhang, Yogesh Sharma, Bethany X. Rutherford, Samyak Dhole, Pinku Roy, Zhehui Wang, Yiquan Wu, Haiyan Wang, Aiping Chen, Quanxi Jia

**Affiliations:** ^1^ Department of Materials Design and Innovation University at Buffalo Buffalo NY 14260 USA; ^2^ Center for Integrated Nanotechnologies (CINT) Los Alamos National Laboratory Los Alamos NM 87545 USA; ^3^ Sandia National Laboratories Albuquerque NM 87185 USA; ^4^ Kazuo Inamori School of Engineering New York State College of Ceramics Alfred University Alfred NY 14802 USA; ^5^ Department Electrical and Computer Engineering School of Materials Engineering Purdue University West Lafayette IN 47907 USA; ^6^ Physics Division Los Alamos National Laboratory Los Alamos NM 87545 USA

**Keywords:** epitaxial films, high‐entropy perovskite oxides, optical energy transfer, thin films

## Abstract

Mixtures of Ce‐doped rare‐earth aluminum perovskites are drawing a significant amount of attention as potential scintillating devices. However, the synthesis of complex perovskite systems leads to many challenges. Designing the A‐site cations with an equiatomic ratio allows for the stabilization of a single‐crystal phase driven by an entropic regime. This work describes the synthesis of a highly epitaxial thin film of configurationally disordered rare‐earth aluminum perovskite oxide (La_0.2_Lu_0.2_Y_0.2_Gd_0.2_Ce_0.2_)AlO_3_ and characterizes the structural and optical properties. The thin films exhibit three equivalent epitaxial domains having an orthorhombic structure resulting from monoclinic distortion of the perovskite cubic cell. An excitation of 286.5 nm from Gd^3+^ and energy transfer to Ce^3+^ with 405 nm emission are observed, which represents the potential for high‐energy conversion. These experimental results also offer the pathway to tunable optical properties of high‐entropy rare‐earth epitaxial perovskite films for a range of applications.

## Graphical abstract

This work describes the synthesis of high‐entropy epitaxial perovskite oxide films composed of multiple luminescent centers Gd^3+^ and Ce^3+^. Such materials exhibit the ability of UV to visible light energy transfer. The experimental results offer a pathway to tunable optical properties for a range of applications.

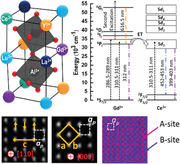

## 1 Introduction

Scintillating materials have found a wide variety of applications in nuclear research, medical imaging, security screening, and high‐energy physics applications.^[^ ^1^, ^2^, ^3^ ^]^ Sought‐after properties often consist of high‐atomic number (Z) materials with high light yield and fast decay times, as well as high energy resolution.^[^ ^1^, ^4^, ^5^, ^6^ ^]^ Rare‐earth (RE) aluminum oxide perovskites doped with Ce^3+^ and similar dopants such as Eu^3+[^ ^7^ ^]^ and Tb^3+[^ ^8^, ^9^ ^]^ have shown promise as scintillating and phosphorescent materials throughout the past 20+ years.^[^ ^1^, ^2^, ^10^, ^11^, ^12^ ^]^ For instance, Ce‐doped LuAlO_3_ (Ce:LuAlO_3_) has a relatively high Z value and has been considered for positron emission tomography due to the high stopping power required for γ‐ray detection.^[^ ^1^, ^5^ ^]^ Ce‐doped YAlO_3_ (Ce:YAlO_3_), which exhibits peculiar pulse height discrimination properties, has also recently been studied for its ability to distinguish between *γ* and neutron radiations.^[^ ^13^ ^]^ To develop scintillators with improved performance across different applications, further investigation of novel scintillating materials is needed.

Ce^3+^ is a common luminescent center among RE‐based perovskite metal oxides and other activated scintillating materials^[^ ^3^, ^5^, ^6^ ^]^ due to the efficient 5d → 4f transition with characteristic ultraviolet to blue visible light emission.^[^ ^12^, ^14^ ^]^ The amount of Ce dopant can greatly affect the performance of materials. For instance, increasing the Ce‐doping concentration in YAlO_3_ could be used to improve the decay time, energy resolution and light yield.^[^ ^15^ ^]^ Doping from 1% to 5% in Ce:LaF_3_ can generally minimize transfer losses.^[^ ^16^ ^]^ Further increasing the Ce composition has been shown to shift the absorption spectra from ultraviolet to the visible range as observed in 20% Ce‐doped Ce:LaAlO_3_.^[^ ^2^ ^]^ Experimental results also illustrated that doping with multiple luminescent centers can exhibit energy transfer among RE^3+^ elements of Gd, Ce, Tb, and Eu and allow for a tunable emission spectrum.^[^ ^17^, ^18^, ^19^ ^]^ Moreover, such mixed rare‐earth crystals can show improved scintillation efficiency and are the material of choice for the brightest scintillators.^[^ ^20^, ^21^ ^]^ This is partially related to the decrease of the thermalization length during the initiation of the ionizing track.^[^ ^16^ ^]^ In the scintillating process, following the absorption of ionizing energy and creation of electron–hole pairs (conversion efficiency), the energy is transferred along an ionizing track to luminescent centers (transfer efficiency), at which point recombination occurs emitting a photon (quantum efficiency).^[^ ^5^, ^6^, ^16^ ^]^ In this regard, mixed crystals may shorten the track length to a luminescent center and possibly result in a faster decay time or short afterglow.

Designing mixed crystals such as mixtures of RE perovskite metal oxides can be challenging. Successful growth, however, can be rewarding with potentially much‐improved efficiency and tunability.^[^ ^16^, ^22^, ^23^ ^]^ For example, altering the concentrations of multiple A‐site cations in perovskite ABO_3_ compounds has been shown to alter the bandgap and modify trap levels. Such phenomena can be observed through shifts in thermally stimulated luminescence (TSL) spectra.^[^ ^24^ ^]^ Bandgap and trap‐level engineering can be beneficial for much‐improved transfer efficiency. The system, however, can be very complex as the trapping of luminescent centers (hole/electron) shows opposite TSL shifting with respect to the altered A‐site concentrations as well as variation in the ratio of electric/magnetic dipole transitions.^[^ ^25^ ^]^ Additionally, mixing can cause disordering within the lattice in the form of antisite defects which can lead to new trap centers and potentially slower decay times.^[^ ^22^, ^23^ ^]^ On the other hand, the disorder has been shown to dramatically increase light yield with relatively lower afterglow while maintaining a high effective atomic number.^[^ ^23^, ^26^, ^27^, ^28^ ^]^ The structural disorder in these materials arises from the cation competition in occupancy of the same lattice location.^[^ ^28^ ^]^ As a result, the differences in atomic radii can lead to large distortion in the lattice that may potentially modify the conduction zone and result in an increased conversion efficiency.^[^ ^23^, ^26^ ^]^ The disorder can also give rise to additional thermalization channels that allow for increased transfer efficiency.^[^ ^28^ ^]^ It has been argued that a compositionally disordered but isovalent cation sublattice could retain anion spatial symmetry with long‐range order, which can lead to improved scintillation properties.^[^ ^29^ ^]^

Entropy‐stabilized oxides (ESOs) allow the design of compositional disorder of a sublattice and have been seen to exhibit a range of desired properties.^[^ ^30^, ^31^ ^]^ Recently, ultralow thermal conductivity was achieved by designing five transition metals in the B‐site of a BaTiO_3_/SrTiO_3_‐based perovskite.^[^ ^32^, ^33^ ^]^ Reduced thermal conductivity has also been observed in ESOs via local ionic charge disorder with no compromise in mechanical stiffness.^[^ ^34^ ^]^ There is an immense amount of high entropy oxide materials (200+) that may show interesting optical properties.^[^ ^35^, ^36^, ^37^, ^38^, ^39^, ^40^, ^41^ ^]^ For example, the stabilization of a high entropy sesquioxide ceramic phosphor has been shown to exhibit multi‐wavelength emission with a high degree of optical transparency in the visible region.^[^ ^42^ ^]^ On the other hand, Ce‐doped RE‐based oxides have been observed to have narrow band gaps with absorption over the entire visible spectra.^[^ ^36^, ^39^ ^]^ Additionally, RE high entropy oxides with multiple optically active cations have shown improved photocatalytic performance when compared to similar single fluorite and equivalent mixed oxides.^[^ ^41^ ^]^

Although high entropy materials design may allow for stabilization with more compositional tuning, achieving a single‐phase with multiple stoichiometry materials can often be challenging.^[^ ^37^ ^]^ Furthermore, the growth of complex perovskite oxides, particularly when a high growth temperature is used to achieve high crystallinity materials, can result in antisite defects, impurities, as well as oxygen vacancies that can degrade crystalline quality. Defects can form deep electron traps and result in degraded optical performance due to undesirable strong UV emission bands.^[^ ^2^, ^43^ ^]^ On the other hand, the perovskite framework offers a variety of functionalities resulting from its rich compositional diversity.^[^ ^44^ ^]^ One way to access single‐phase perovskite metal oxides with complex chemical compositions is with pulsed laser deposition (PLD). Experimental results have shown that PLD can be used to grow epitaxial high entropy perovskite metal oxide films.^[^ ^33^ ^]^ The ability to stabilize perovskites with the configurational disorder in such high entropy materials could offer even more new opportunities for functionalities not available from the conventional perovskite metal oxide.^[^ ^33^, ^37^ ^]^

Much like the bulk crystals, ceramics and glasses have dominated the scintillating materials. These materials in film form have also been explored because of their greater spatial resolution and smaller light attenuation resulting directly from their lesser thickness (≈10–25 µm) when compared to single crystals (≈0.5–1 mm).^[^ ^15^, ^43^, ^45^, ^46^, ^47^, ^48^ ^]^ For instance, Eu‐doped Lu_2_O_3_ film (10 µm) has shown significant sensitivity with brighter ionization tracks for radioluminescence microscopy of human cancer cells in comparison with conventional CdWO_4_ (500 µm) crystals.^[^ ^45^ ^]^ Furthermore, epitaxial Eu:(Gd,Lu)AlO_3_ film also exhibits better contrast performance than the currently used Eu:Gd_3_Ga_5_O_12_ or Tb:Lu_2_SiO_5_ for high‐resolution hard X‐ray imaging. Ce:(Gd,Lu)AlO_3_ has shown to have a decay time of ≈50 ns that could be used for ultra‐fast imaging.^[^ ^46^ ^]^

In this work, we report the growth of rare‐earth high entropy perovskite oxide (REHEPO) thin films (≈147 nm in thickness) and discuss their structural and optical properties. The films have equal concentrations of RE cations (5RE^3+^_0.2_)AlO_3_ and are of a single phase (La_0.2_Lu_0.2_Y_0.2_Gd_0.2_Ce_0.2_)AlO_3_ epitaxially grown on LaAlO_3_ (LAO) substrates using PLD. This material system displays luminescence of the active A‐site cations and demonstrates the ability of UV to visible light energy transfer with further potential use for downgraded emission and high‐energy applications. The new material offers the ability for designing the A‐site cations and controlling wavelength emission with much less challenging synthesis than mixed crystal scintillators, while also maintaining the additional benefits associated with a highly crystalline lattice. X‐ray diffraction (XRD) and scanning transmission electron microscopy (STEM), including energy dispersive X‐ray spectroscopy (EDS), high‐angle annular dark‐field (HAADF) and bright‐field (BF) imaging, were used to characterize the microstructure of the films. Ultraviolet–visible (UV–Vis) spectroscopy and photoluminescent (PL) spectroscopy were conducted to analyze the bandgap, absorption, transmittance, excitation, and emission spectra of the REHEPO thin films.

## 2 Results and Discussion

### 2.1 X‐Ray Diffraction of Highly Crystalline REHEPO Film

**Figure 1:**
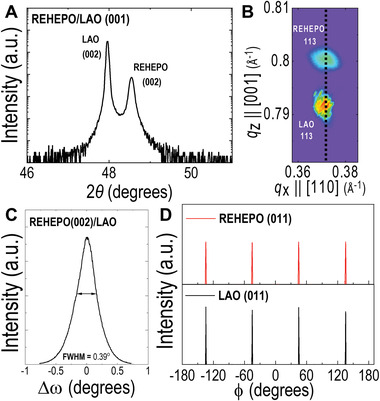
X‐ray characterization of REHEPO/LAO heterostructure. a) Local 2*θ*‐*ω* scan of REHEPO thin film grown on LAO (001) substrate. b) RSM around the (113) reflection showing the strained film on LAO substrate. c) Rocking curve *ω*‐scan of the (002) peak of the film. d) *ϕ*‐scan of REHEPO (011) and (011) LAO substrate.

High crystallinity epitaxial REHEPO films are confirmed from XRD measurements. **Figure** **1** shows the XRD results of REHEPO thin film grown on LaAlO_3_ (001) substrates. The local 2*θ*‐*ω* scan shown in Figure 1a indicates the formation of a single phase REHEPO film with (002) orientation (not shown are the peaks from (001) and (003) located at 23.72° and 76.14°, respectively, as shown in Figure S1, Supporting Information). These peak locations coincide well with reported peaks for the individual RE aluminates that compose the film.^[^ ^49^, ^50^, ^51^, ^52^ ^]^ The rocking curve of REHEPO (002) shows a full width at half maximum (FWHM) of 0.39° indicating a high crystallinity film (Figure 1c). Using Scherrer equation, we estimated the mean size of crystallites to be around 90 nm. Figure 1d shows the *ϕ*‐scans of REHEPO (011) and LAO (011). Four peaks at 90° apart from each other indicate four‐fold symmetry of the REHEPO film epitaxially grown on LAO substrate. The film has an average in‐plane mosaic of 0.32°_,_ which is 0.10° greater than the single‐crystal LAO substrate.

The lattice mismatch between the REHEPO thin film (*a*_f_ = 3.735 Å) and substrate (*a*_s_ = 3.792 Å) is 1.51%, which indicates a likely in‐plane tensile strain for a pseudo‐cubic REHEPO unit cell. The pseudo‐cubic lattice parameter is calculated by averaging all individual aluminates.^[^ ^49^, ^50^, ^51^, ^53^, ^54^ ^]^ Using the thin film (002) peak, we have calculated the out‐of‐plane pseudo‐cubic lattice parameter *c* = *a_oop_* = 3.756 Å. By using this *c‐*lattice parameter and (011) diffraction from the tilted 2*θ*‐*ω* scan, we can calculate the in‐plane pseudo‐cubic lattice parameter of *b* = *a_ip_* = 3.795 Å. The results are consistent with those calculated from the reciprocal space mapping (RSM) around (113) of the REHEPO thin film and LAO substrate (Figure 1b). Based on the RSM results shown in Figure 1b, the out‐of‐plane and in‐plane pseudo‐cubic lattice parameters can be calculated as *a_oop_* = 3.748 Å and *a_ip_* = 3.795 Å, respectively. These parameters are calculated by taking the scalar projection of the film (113) onto either *q_x_* [110] or *q_z_* [001] directions (*Proj*_[110]_^(113)^ = $\sqrt{2}$, *Proj*_[001]_^(113)^ = 3), and then dividing by the corresponding inverse *d*‐spacing coordinate of the film peak (*q_x_ =* 0.3727 Å^−1^, *q_z_ =* 0.8005 Å^−1^) as detailed in the Figure S2, Supporting Information. A tensile strain for both in‐plane and out‐of‐plane directions may suggest that a more detailed analysis is necessary to evaluate the effective lattice parameter of REHEPOs. This is conceivable by considering that REHEPO films may have cubic, tetragonal, hexagonal, orthorhombic or rhombohedral structures that are related to the Goldschmidt tolerance factor. The effective ionic radii for most RE cations with a 3+ oxidation state and coordination number N < 9 have been reported by Shannon and Prewitt.^[^ ^55^ ^]^ On the other hand, the coordination number of perovskite cations depends on both the site location and the structure. For example, the A‐site of cubic perovskite has a coordination number *N* = 12 and the Shannon radii database is not complete for RE‐based perovskites. As such, we used the reported effective ionic radii based on the data‐driven method that is termed as a sure independence screening and sparsifying operator (SISSO).^[^ ^56^, ^57^ ^]^ This method has been used to extend the Shannon radii database and is consistent with other previously reported estimates.^[^ ^58^ ^]^ Using the obtained values from the SISSO extended database, our calculated tolerance factor, *t* = 0.97, indicates that REHEPO should be a slightly distorted orthorhombic following the Goldschmidt tolerance parameter rules for an ideal cubic perovskite (A‐site has 12‐coordination, B‐site has 6‐coordination).^[^ ^40^, ^53^, ^59^ ^]^ More discussion about the crystal structure of the REHEPO films will be presented in the next section. We would also like to point out that the formation of single phase REHEPO film in our design is favorable, considering that the atomic‐size difference, *δ*(*R_A_*), is about 6.56%, although limited experimental results have shown that the cation‐size difference does not appear to be an important factor for the formation of single high‐entropy perovskite phases.^[^ ^60^ ^]^

### 2.2 High‐Resolution Electron Microscopy Characterization of Orthorhombic Lattice Structure

**Figure 2:**
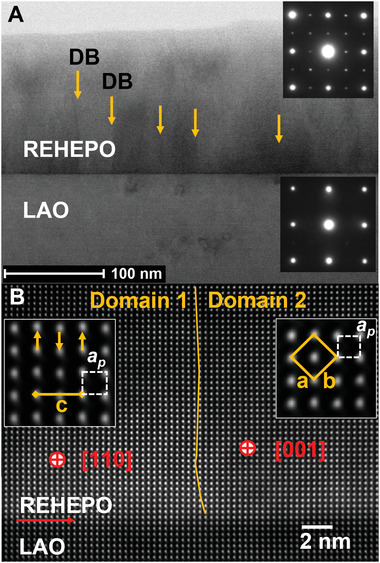
Structural characterization of a REHEPO film by STEM: a) A STEM BF image taken along LAO [100] axis, showing the REHEPO film on LAO substrate, along with SAEDs (insets) from the film and substrate, respectively. Within the REHEPO film, there are three equivalent orientations or domains, corresponding to its *c*‐axis parallel to *a*‐, *b*‐, and *c*‐axes of LAO substrate, respectively. The domain boundaries (DBs) in the REHEPO films are marked by vertical arrows. b) A high‐resolution STEM HAADF image showing the atomic structure of the DB and REHEPO/LAO interface. Insets in (b) are highly magnified STEM images displaying the atomic structure of REHEPO in [110] projection (domain 1) and REHEPO in [001] projection (domain 2), with respective unit cells marked (*a_p_*,perovskite pseudo‐cubic cell and *a*, *b*, and *c*, lattice constants of the orthorhombic cell).

The structure of the REHEPO film grown on LAO was studied by cross‐sectional STEM. **Figure** **2**a shows STEM BF image taken in LAO [100] axis, along with the respective selected area electron diffraction (SAED) patterns (insets) from the REHEPO and LAO substrate. The SAED from the REHEPO is nearly identical to that of LAO, except for the presence of weak superlattice reflections, indicating the REHEPO is a perovskite‐like structure and epitaxially grown on the substrate. The careful analysis found that the REHEPO has a structure of GdFeO_3_ or SrRuO_3_, which has an orthorhombic unit cell resulting from the slight monoclinic distortion of the cubic cell. The orthorhombic cell has *a*, *b* of about $\sqrt{2}$ *a_p_* and *c* of 2*a_p_*, where *a_p_* is the perovskite pseudo‐cubic cell constant. With such a structure, there are three equivalent domains in the film, corresponding to the *c*‐axis of the domain parallel to [100], [010], and [001] axis of LAO substrate, respectively. The domain boundaries are visible in Figure 2a as marked by vertical arrows. The resulting SAED pattern with the superlattice reflections is consistent with the orthorhombic cell.^[^ ^61^ ^]^ Figure 2b shows a high‐resolution HAADF image of REHEPO/LAO interface with a vertical domain boundary (DB) within the REHEPO. The highly magnified insets in Figure 2b show the detailed structure of the REHEPO domains in two perpendicular projections. Domain 1 is in [110] projection, giving rise to the *c*‐axis in the horizontal direction. The small relative displacement in the vertical direction (marked by arrows in the left inset Figure 2b) leads to a doubling of the unit cell constant or *c* = 2*a_p_*. Domain 2, on the other hand, is in [001] projection. The relationship between the *a*, *b* axes of the orthorhombic cell and the pseudo‐cubic cell (*a_p_*) in this projection can be clearly seen in the right inset in Figure 2b. In reference to the pseudo‐cubic cell, the film grows in a cube‐on‐cube epitaxial relationship with respect to LAO. The measurements from the high‐resolution STEM images show the orthorhombic cell lattice constants *a* and *b* of about 5.33 Å, *c* of about 7.57 Å and the pseudo‐cubic cell constant *a_p_* of about 3.785 Å.

**Figure 3:**
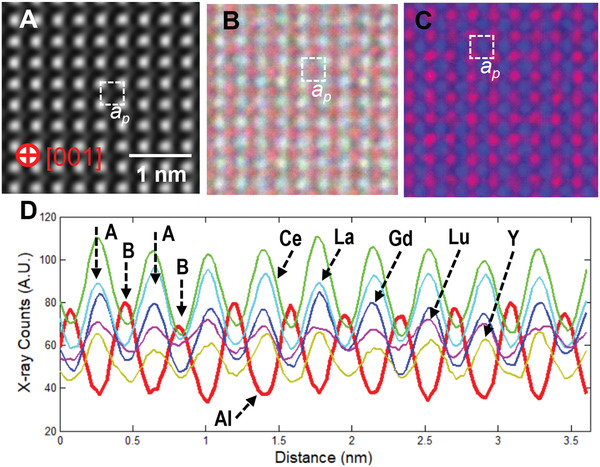
Atomic‐scale EDS mapping of REHEPO structure: a) A STEM‐HAADF image of the film in [001] projection. b) A composite EDS map, made of Al K—red, Ce L—green, Gd L—blue, La L—cyan, Lu L—magenta, and Y L + K—yellow. c) A composite EDS map, made of Al K in blue and the sum of Ce L, Gd L, La L, Lu L, and Y L + K in red. d) EDS line profiles (Al K—red, Ce L—green, Gd L—blue, La L—cyan, Lu L—magenta, and Y L + K—yellow) from (b) along the horizontal direction. In (d), the positions of A‐ and B‐sites in perovskite ABO_3_ pseudo‐cubic cell are marked by the arrows. The colors represented here in (d) are consistent with the colors described for the composite EDS map in (b).

It is interesting to understand the distribution of high entropy elements within the REHEPO structure. This was achieved by atomic‐scale EDS mapping as shown in **Figure** **3**. The EDS mapping was done along the [001] REHEPO domain (Figure 3a). The EDS maps (Figure 3b,c) and line‐profile (Figure 3d) show that the element Al occupies B‐sites, while Y and RE elements (La, Ce, Gd, Lu) occupy A‐sites in the perovskite pseudo‐cubic (*a_p_*) ABO_3_ cell. Furthermore, the line‐profile also indicates that Y and RE elements (La, Ce, Gd, Lu) are randomly distributed on A sites or without obvious ordering. Additionally, we have conducted electron energy loss spectroscopy (EELS) to determine the oxidation state of Ce in our samples (Figure S3, Supporting Information). We have approximated the valence state of Ce in our thin films around 3 using the integral area ratio (R) method^[^ ^62^ ^]^ for the M‐edge double lines (M5/M4) attributed to Ce.

### 2.3 UV–Vis and PL Analysis of Optical Energy Transfer

**Figure 4:**
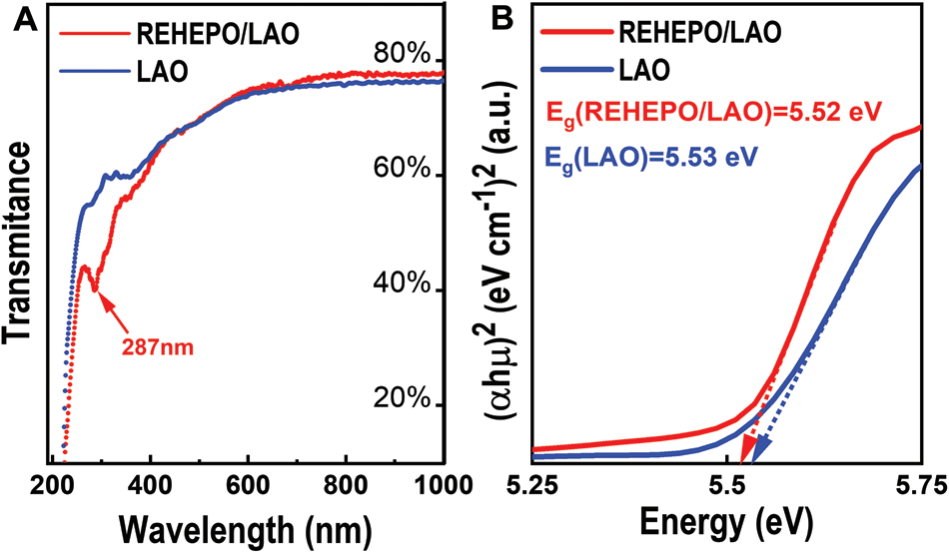
UV–Vis spectroscopy of the REHEPO/LAO heterostrucutre compared to LAO substrate. a) Transmittance and b) Tauc plot of the thin film and substrate.

**Figure 5:**
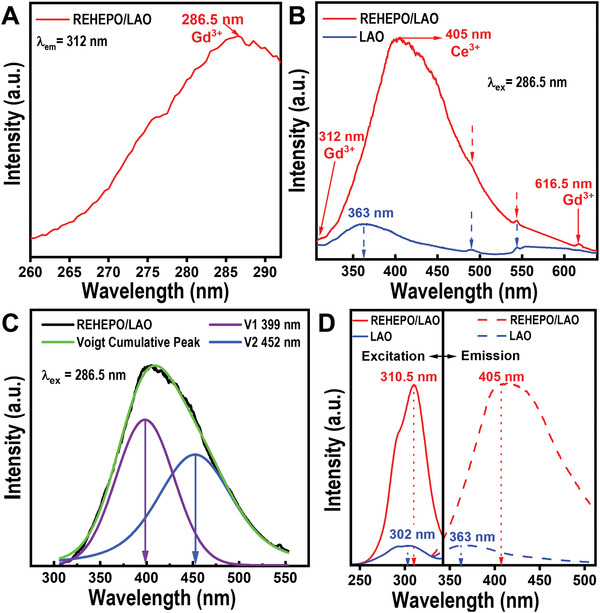
Photoluminescence results of a) excitation spectra of Gd^3+^ from the REHEPO thin film with characteristic emission of 312 nm and b) emmission spectra of Ce^3+^ from the REHEPO/LAO heterostructure and LAO substrate at 286.5 nm excitation. The blue arrows indicate emission peaks associated with the LAO substrate which, also appear in the REHEPO/LAO heterostructure and can be seen as dashed red arrows. c) Deconvoluted Voigt fitting of Ce^3+^ 405 nm emission from the REHEPO/LAO heterostructure with two components of max intensity V1 = 399 nm and V2 = 452 nm. d) Photoluminescnce spectra of the REHEPO/LAO heterstructure and LAO substrate with characteristic emission (*λ*_em_ = 405 nm) and excitation (*λ*_ex_ = 310.5 nm).

The optically active Gd^3+^ and Ce^3+^ inherent to the REHEPO films make such materials attractive for phosphorescent applications. In this regard, UV–Vis and PL spectroscopy measurements were conducted and the results are shown in **Figures** **4** and **5**. The REHEPO/LAO heterostructure shows 65–78% transmittance for the visible and infrared spectra. The REHEPO thin film shows a signature peak in both transmittance and absorption spectra at 287 nm in the UV range (Figure 4a and Figure S4, Supporting Information). The thin film also shows only a slight difference in bandgap energy (5.52 eV) in comparison with the LAO substrate, which is consistent with Ce:LaAlO_3_ shown in Figure 4b.^[^ ^63^ ^]^

Excitation spectra with characteristic emission of 312 nm can be seen in Figure 5a, where the maximum intensity at 286.5 nm corresponds to the ^6^P_J_ → ^8^S_7/2_ transition in Gd^3+^. Our observation is consistent with the signature peak seen in the UV–Vis spectra above and reported in the literature.^[^ ^17^, ^21^, ^42^, ^64^ ^]^ As can be seen from Figure 5b, the intensity of the REHEPO/LAO heterostructure is much stronger than that of the substrate alone. This indicates that the luminescence comes mainly from the REHEPO film with little or negligible contribution from the substrate. The REHEPO thin film has the greatest intensity at 405 nm under excitation of 286.5 nm, which can be an indication of luminescence from the 5d → 4f transition found in the Ce^3+^ that has been redshifted.^[^ ^2^, ^14^, ^17^, ^21^ ^]^ Given the asymmetric nature of the photoluminescence spectrum shown in Figure 5c, we have used Voigt fitting to deconvolute the 405 nm emission peak. The two well‐defined peaks with Voigt distribution at maximum intensities of 399 and 452 nm can be attributed to the ^5^D_J_ → ^2^F_5/2_ and ^5^D_J_ → ^2^F_7/2_ transitions, respectively. Since the excitation at 286.5 nm is characteristic of Gd^3+^ and the emission of 405 nm is characteristic of Ce^3+^, one can argue that there exists an energy transfer (ET) from Gd^3+^ to Ce^3+^ via the ^6^P_J_ → ^5^D_J_ transition and emission at 405 nm via the ^5^D_J_ → ^2^F_J_ transition.

**Figure 6:**
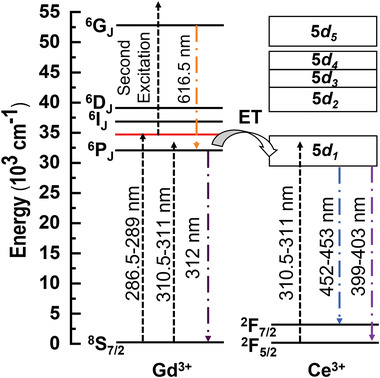
Energy level diagram of Gd^3+^ and Ce^3+^ in REHEPO thin film with excitation/emission mechanims showing energy transfer (ET). The red line indicates a virtual state part of a possible two‐photon excitation process.

The photoluminescence spectra seen in Figure 5d summarize the excitation and emission spectra with characteristic emission (*λ*_em_ = 405 nm) and excitation (*λ*_ex_ = 310.5 nm). Deconvolution of the excitation spectra reveals two modes of excitation with the stronger peak at ≈311 nm (Figure S5, Supporting Information). The lower intensity deconvoluted Voigt peak is found to be ≈289 nm which corresponds to a forbidden or virtual state within the Gd^3+^ optical energy levels. Unlike the 311 nm wavelength, excitation energy at 289 nm may be less likely to transfer energy to the 5d orbital of Ce^3+^ and instead can result in the 312 nm emission seen in Figure 5a. However, this excitation energy at 289 nm can still lead to the ET as previously discussed and be part of a two‐photon process with longer wavelength emission observed in Gd^3+^. Figure 5b shows a low intensity longer wavelength emission peak of 616.5 nm that corresponds to the ^6^G_J_ → ^6^P_J_ transition in Gd^3+^ and is likely the resulting emission of a two‐photon process.^[^ ^64^, ^65^ ^]^ Luminescence from this transition in Gd^3+^ may be due to the combination of two photons with wavelengths of 289 nm that create an exciton from the ground state, ^8^S_7/2_, reaching above or within ^6^G_J_ energy levels before recombining and emitting the observed 616.5 nm peak. The characteristic ^6^P_J_ → ^8^S_7/2_ transition within Gd^3+^ can be considered energy loss or inefficiencies with respect to the most desirable 405 nm emission. It is also possible that photons at the wavelength of 310.5 nm are able to excite Ce^3+^ leading to the emission at 405 nm.^[^ ^21^ ^]^ Along with the ET from Gd^3+^_,_ this may indicate multiple thermalization pathways to the 405 nm emission. An illustration of all excitation/emission mechanisms is visualized in **Figure** **6** and additional deconvoluted spectra can be found in Figure S5, Supporting Information.

## 3 Conclusion

The synthesis of high‐entropy perovskite oxide thin films composed of multiple luminescent centers, Gd^3+^ and Ce^3+^, has enabled us to design materials with combined intrinsic luminescent properties. The use of high Z elements such as Lu^3+^, Y^3+^, and La^3+^ in our REHEPOs can increase the overall density and facilitate the access to the fast decay times and peak discrimination properties observed similarly in perovskite aluminate‐based scintillating devices.^[^ ^1^, ^5^, ^13^ ^]^ Our results demonstrate that a high entropy oxide system with equiatomic A‐site cations allows for the achievement of desired optical luminescence observed in mixtures and/or doping of RE aluminates while maintaining a highly crystalline single‐phase. Atomic‐scale EDS mapping shows that the A‐site cations appear to be randomly distributed with proper separation between B‐site cations indicating negligible prevalent antisite defects. The high crystallinity of these materials paves the way for exciting new research of high precision scintillating applications. Further tuning of the A‐site cations may potentially change the unit cell structure and lattice parameters allowing for alternative matching with other substrates. Additionally, strain‐engineering through the use of alternative substrates and nanocomposite epitaxial relationships may allow for further tuning of structural and optical properties.^[^ ^44^, ^66^, ^67^, ^68^ ^]^

We would also like to emphasize that there exists the possibility of an optical cascade for downgraded emission. As discussed previously,^[^ ^17^, ^18^, ^19^ ^]^ energy transfer for Ce^3+^ → Tb^3+^ → Eu^3+^ can occur. In our REHEPO/LAO system, energy transfer via Gd^3+^ → Ce^3+^ may lead to a potential optical cascade of Gd^3+^ → Ce^3+^ → Tb^3+^ → Eu^3+^ and the resulting light conversion tunability of UV → blue → green → red depending on luminescent elements added to the A‐site cation. The highly disordered lattice, on the other hand, may lead to shortened ionization tracks for faster decay times. Furthermore, detection of *α*‐particle or X‐ray radiation may be possible when considering the high atomic number of Lu that allows for greater absorption of high energy radiation. The highly epitaxial films could also provide additional benefits in terms of optimal spatial resolution. We believe that further exploitation of the A‐site cations in REHEPO could allow for a tunable emission phosphor with applications for highly efficient scintillating devices. This work also helps to extend the optical research of high entropy oxide thin films into the field of high‐energy applications.

## 4 Experimental Section

### PLD Thin Film Synthesis

The stoichiometric target (La_0.2_Lu_0.2_Y_0.2_Gd_0.2_Ce_0.2_)AlO_3_ provided by Plasmaterials was used for the deposition after re‐sintering at 1500 °C and surface polishing with coarse grit sandpaper. A Coherent, Inc. KrF excimer laser (*λ* = 248 nm) was operated at a pulse frequency of 5 Hz and laser fluence of 2.00 J cm^−2^ for the deposition of the films. Single‐sided polished LaAlO_3_ (001) supplied by MTI was used as the substrate. The base pressure of the chamber was 4.5 × 10^−7^_ _Torr before introducing O_2_ for thin film deposition. The substrate temperature was initially optimized and maintained at 775 °C during the growth of the films. The O_2_ pressure was kept at 3 × 10^−5^ Torr. The films were immediately quenched at an oxygen pressure of 1.1 Torr O_2_ after the growth.

### XRD Characterization

A PANalytical X'Pert Pro X‐ray diffractometer equipped with Cu K*α* (*λ* = 1.54184 Å) source was used for 2*θ*–*ω*, rocking curve, *ϕ* and RSM scans. A hybrid monochromator 2xGe (220) with fixed divergence slit of 1/8″ was used for the incident beam path and Ni beam attenuator of 0.125 mm with an asymmetrical analyzer for the diffracted beam path was used for all measurements. RSM measurements were conducted using a PIXcel3D‐Medipix3 area detector for the diffracted beam path. All samples were first aligned, where the diffraction peaks from LAO substrate were used as the reference.

### STEM Structural Characterization

An FEI Titan G2 80–200 STEM with a Cs probe corrector and ChemiSTEM technology (X‐FEG and SuperX EDS with four windowless silicon drift detectors), operated at 200 kV, was used in this study. STEM imaging, atomic‐scale EDS elemental mapping and SAED were used to determine the structures of REHEPO films on LAO substrates. For atomic‐scale chemical mapping, EDS spectral image data were acquired with an electron probe of size <0.15 nm, convergence angle of 18.1 mrad, and current of ≈100 pA. EELS (GATAN 963) was used to probe the Ce fine structure of the films under similar optical conditions with energy dispersion 0.25 eV/channel, and an instantaneous dwell time of 500 ms. STEM HAADF and bright‐field (BF) images were recorded under similar optical conditions using an annular detector with a collection range of 60–160 mrad and 0–30 mrad, respectively.

### UV–Vis and PL Characterization

Real in‐line transmittance and absorption spectra were recorded with a UV–Vis Spectrophotometers (Evolution 220, Thermo Scientific) from 190 to 1100 nm. Photoluminescence excitation and emission spectra were measured with a Fluorolog‐3 modular spectrofluorometer (Jobin Yvon Inc., Horiba).

### Statistical Analysis

Original RSM data was transformed from 2*θ*‐*ω* space to *q_x_* − *q_z_* reciprocal space and the log of the intensity was used for RSM measurement seen in Figure 1b. The thin film peak inverse *d*‐spacing coordinates were obtained from interpolated RSM data using the SciPy library^[^ ^69^ ^]^ function grid data with the nearest neighbor interpolation seen in Figure S2, Supporting Information. The average *q_x_* and *q_z_* coordinates of the interpolated maximum values were used to calculate the inverse *d*‐spacing coordinates of the film peak with a standard deviation of ± 0.00281Å^−1^ ± 0.00124 Å^−1^ for *q_x_* and *q_z_* values, respectively. The optical band gap of samples was estimated with a Tauc plot converted from the measured absorption spectra seen in Figure 4. OriginLab was used for peak fitting and deconvolution of PL measurements in Figure 5c and Figure S5, Supporting Information as well as for peak fitting and FWHM calculations in Figure 1b,c. EDS spectral images were acquired as a series of frames, where the same region was scanned multiple times. Frames were spatially drift‐corrected to build up spectral image data.^[^ ^70^ ^]^ Elemental maps of Al K, Gd L, Lu L, and Y L & K were extracted from the spectral image with selected EDS energy widows for each element. Due to the significant overlapping of La L and Ce L X‐ray lines, maps of La L and Ce L were carried out after the pixel‐by‐pixel deconvolution using La K and Ce L reference spectrum obtained from LAO and CeO_2_, respectively.

## Conflict of Interest

The authors declare no conflict of interest.

## Supporting information

Supporting Information

## Data Availability

The data that support the findings of this study are available from the corresponding author upon reasonable request.

## References

[1] A. Lempicki , J. Glodo , Nucl. Instrum. Methods Phys. Res., Sect. A 1998, 416, 333.

[2] J. Pejchal , J. Barta , T. Trojek , R. Kucerkova , A. Beitlerova , M. Nikl , Radiat. Meas. 2019, 121, 26.

[3] C. Dujardin , E. Auffray , E. Bourret‐Courchesne , P. Dorenbos , P. Lecoq , M. Nikl , A. N. Vasil'ev , A. Yoshikawa , R.‐Y. Zhu , IEEE Trans. Nucl. Sci. 2018, 65, 1977.

[4] J. A. Mareš , N. Čechová , M. Nikl , J. Kvapil , R. Krátký , J. Pospíšil , J. Alloys Compd. 1998, 275–277, 200.

[5] D. S. McGregor , Annu. Rev. Mater. Res. 2018, 48, 245.

[6] C. L. Melcher , J. Nucl. Med. 2000, 41, 1051. 10855634

[7] P. J. Dereń , J. C. Krupa , J. Lumin. 2003, 102–103, 386.

[8] X. Liu , L. Yan , J. Lin , J. Phys. Chem. C 2009, 113, 8478.

[9] P. Zhang , M. Gao , R. Guo , J. Xu , Y. Wang , L. Luo , Optik 2021, 239, 166880.

[10] L. Lu , M. Sun , T. Wu , Q. Lu , B. Chen , B. Huang , Nanoscale Adv. 2022, 4, 680. 36131822 10.1039/d1na00815c PMC9417099

[11] V. Gorbenko , T. Zorenko , K. Paprocki , F. Riva , P. A. Douissard , T. Martin , Y. Zhydachevskii , A. Suchocki , A. Fedorov , Y. Zorenko , CrystEngComm 2018, 20, 937.

[12] V. G. Baryshevskyt , M. V. Korzhikt , B. I. Minkovt , A. Smimovat , A. A. Fyodorovt , P. Dorenbost , J. Phys.: Condens. Matter 1993, 5, 7893.

[13] L. Viererbl , V. Klupak , M. Vins , Z. Lahodova , J. Soltes , IEEE Trans. Nucl. Sci. 2016, 63, 1963.

[14] O. Sidletskiy , P. Arhipov , S. Tkachenko , I. Gerasymov , G. Trushkovsky , T. Zorenko , Y. Zorenko , P. Mateychenko , A. Puzan , W. Gieszczyk , P. Bilski , Crystals 2019, 9, 296.

[15] K. Nakanishi , S. Yamamoto , K. Kamada , A. Yoshikawa , Appl. Radiat. Isot. 2021, 168, 109483. 33323313 10.1016/j.apradiso.2020.109483

[16] A. V. Gektin , A. N. Belsky , A. N. Vasil'ev , IEEE Trans. Nucl. Sci. 2014, 61, 262.

[17] A. C. Yanes , J. del‐Castillo , E. Ortiz , J. Alloys Compd. 2019, 773, 1099.

[18] P.‐A. Douissard , T. Martin , F. Riva , E. Mathieu , Y. Zorenko , V. Savchyn , T. Zorenko , A. Fedorov , IEEE Trans. Nucl. Sci. 2014, 61, 433.

[19] M. Kucera , M. Rathaiah , A. Beitlerova , R. Kucerkova , M. Nikl , IEEE Trans. Nucl. Sci. 2020, 67, 1049.

[20] O. Sidletskiy , Phys. Status Solidi A 2018, 215, 1701034.

[21] M. Rathaiah , M. Kucera , J. Pejchal , A. Beitlerova , R. Kucerkova , M. Nikl , Radiat. Meas. 2019, 121, 86.

[22] M. Korzhik , A. Gola , J. Houžvička , A. Mazzi , S. Nargelas , S. Sýkorová , G. Tamulaitis , A. Vaitkevičius , Nucl. Instrum. Methods Phys. Res., Sect. A 2019, 927, 169.

[23] M. Korzhik , V. Alenkov , O. Buzanov , A. Fedorov , G. Dosovitskiy , L. Grigorjeva , V. Mechinsky , P. Sokolov , Y. Tratsiak , A. Zolotarjovs , V. Dormenev , A. Dosovitskiy , D. Agrawal , T. Anniyev , M. Vasilyev , V. Khabashesku , Cryst. Res. Technol. 2019, 54, 1800172.

[24] Y. Zhydachevskyy , Y. Hizhnyi , S. G. Nedilko , I. Kudryavtseva , V. Pankratov , V. Stasiv , L. Vasylechko , D. Sugak , A. Lushchik , M. Berkowski , A. Suchocki , N. Klyui , J. Phys. Chem. C 2021, 125, 26698. 10.1021/acs.jpcc.1c06573 PMC8672454 34925675

[25] W. Gieszczyk , A. Mrozik , P. Bilski , V. Vistovskyy , A. Voloshinovskii , K. Paprocki , T. Zorenko , Y. Zorenko , Crystals 2020, 10, 385.

[26] A. Belsky , A. Gektin , A. N. Vasil'ev , Phys. Status Solidi B 2020, 257, 1900535.

[27] M. Pokorný , V. Babin , A. Beitlerová , K. Jurek , J. Polák , J. Houžvička , D. Pánek , T. Parkman , V. Vaněček , M. Nikl , NPG Asia Mater 2021, 13, 66.

[28] M. V. Korzhik , Devices Methods Meas. 2021, 12, 280 (in Russian).

[29] M. Korzhik , A. Fedorov , G. Dosovitskiy , T. Anniyev , M. Vasilyev , V. Khabashesku , Materials 2021, 14, 4889. 34500978 10.3390/ma14174889 PMC8432690

[30] C. M. Rost , E. Sachet , T. Borman , A. Moballegh , E. C. Dickey , D. Hou , J. L. Jones , S. Curtarolo , J.‐P. Maria , Nat. Commun. 2015, 6, 8485. 26415623 10.1038/ncomms9485 PMC4598836

[31] Y. Sharma , M.‐C. Lee , K. C. Pitike , K. K. Mishra , Q. Zheng , X. Gao , B. L. Musico , A. R. Mazza , R. S. Katiyar , V. Keppens , M. Brahlek , D. A. Yarotski , R. P. Prasankumar , A. Chen , V. R. Cooper , T. Z. Ward , ACS Appl. Mater. Interfaces 2022, 14, 11962. 35226475 10.1021/acsami.2c00340

[32] R. Banerjee , S. Chatterjee , M. Ranjan , T. Bhattacharya , S. Mukherjee , S. S. Jana , A. Dwivedi , T. Maiti , ACS Sustainable Chem. Eng. 2020, 8, 17022.

[33] Y. Sharma , B. L. Musico , X. Gao , C. Hua , A. F. May , A. Herklotz , A. Rastogi , D. Mandrus , J. Yan , H. N. Lee , M. F. Chisholm , V. Keppens , T. Z. Ward , Phys. Rev. Mater. 2018, 2, 060404.

[34] J. L. Braun , C. M. Rost , M. Lim , A. Giri , D. H. Olson , G. N. Kotsonis , G. Stan , D. W. Brenner , J. Maria , P. E. Hopkins , Adv. Mater. 2018, 30, 1805004. 10.1002/adma.201805004 PMC9486463 30368943

[35] B. L. Musicó , D. Gilbert , T. Z. Ward , K. Page , E. George , J. Yan , D. Mandrus , V. Keppens , APL Mater. 2020, 8, 040912.

[36] C. Oses , C. Toher , S. Curtarolo , Nat. Rev. Mater. 2020, 5, 295.

[37] A. Amiri , R. Shahbazian‐Yassar , J. Mater. Chem. A 2021, 9, 782.

[38] V. I. Sachkov , R. A. Nefedov , I. V. Amelichkin , IOP Conf. Ser.: Mater. Sci. Eng. 2019, 597, 012005.

[39] R. Djenadic , A. Sarkar , O. Clemens , C. Loho , M. Botros , V. S. K. Chakravadhanula , C. Kübel , S. S. Bhattacharya , A. S. Gandhi , H. Hahn , Mater. Res. Lett. 2017, 5, 102.

[40] A. Sarkar , R. Djenadic , D. Wang , C. Hein , R. Kautenburger , O. Clemens , H. Hahn , J. Eur. Ceram. Soc. 2018, 38, 2318.

[41] S. Nundy , D. Tatar , J. Kojčinović , H. Ullah , A. Ghosh , T. K. Mallick , R. Meinusch , B. M. Smarsly , A. A. Tahir , I. Djerdj , Adv. Sustainable Syst. 2022, 6, 2200067.

[42] G. Zhang , I. Milisavljevic , K. Grzeszkiewicz , P. Stachowiak , D. Hreniak , Y. Wu , J. Eur. Ceram. Soc. 2021, 41, 3621.

[43] M. Buryi , V. Laguta , M. Nikl , V. Gorbenko , T. Zorenko , Y. Zorenko , CrystEngComm 2019, 21, 3313.

[44] S. Dhole , A. Chen , W. Nie , B. Park , Q. X. Jia , Nanomaterials 2022, 12, 835. 35269323 10.3390/nano12050835 PMC8912649

[45] D. Sengupta , S. Miller , Z. Marton , F. Chin , V. Nagarkar , G. Pratx , Adv. Healthcare Mater. 2015, 4, 2064. 10.1002/adhm.201500372 PMC4715786 26183115

[46] F. Riva , P.‐A. Douissard , T. Martin , F. Carlà , Y. Zorenko , C. Dujardin , CrystEngComm 2016, 18, 608.

[47] K. V. Ezirmik , Mater. Res. Express 2021, 8, 016407.

[48] F. Riva , T. Martin , P. A. Douissard , C. Dujardin , J. Instrum. 2016, 11, C10010.

[49] F. Guo , Q. Li , H. Zhang , X. Yang , Z. Tao , X. Chen , J. Chen , Crystals 2019, 9, 245.

[50] H. Asano , S. Kubo , O. Michikami , M. Satoh , T. Konaka , Jpn. J. Appl. Phys. 1990, 29, L1452.

[51] R. W. Simon , C. E. Platt , A. E. Lee , G. S. Lee , K. P. Daly , M. S. Wire , J. A. Luine , M. Urbanik , Appl. Phys. Lett. 1988, 53, 2677.

[52] S. Chaudhury , S. C. Parida , K. T. Pillai , K. D. Singh Mudher , J. Solid State Chem. 2007, 180, 2393.

[53] A. Casu , P. C. Ricci , J. Solid State Chem. 2011, 184, 3028.

[54] A. Chopelas , Phys. Chem. Miner. 2011, 38, 709.

[55] R. D. Shannon , C. T. Prewitt , Acta Crystallogr., Sect. B" Struct. Sci. 1970, 26, 1046.

[56] R. Ouyang , Chem. Mater. 2020, 32, 595.

[57] R. Ouyang , S. Curtarolo , E. Ahmetcik , M. Scheffler , L. M. Ghiringhelli , Phys. Rev. Mater. 2018, 2, 083802.

[58] Y. Q. Jia , J. Solid State Chem. 1991, 95, 184.

[59] N. Ramadass , Mater. Sci. Eng. 1978, 36, 231.

[60] S. Jiang , T. Hu , J. Gild , N. Zhou , J. Nie , M. Qin , T. Harrington , K. Vecchio , J. Luo , Scr. Mater. 2018, 142, 116.

[61] P. Lu , F. Chu , Q. X. Jia , T. E. Mitchell , J. Mater. Res. 1998, 13, 2302.

[62] G. Yang , G. Möbus , R. J. Hand , Micron 2006, 37, 433. 16481178 10.1016/j.micron.2005.12.002

[63] C. N. Singh , G. Pilania , J. Bárta , B. P. Uberuaga , X.‐Y. Liu , J. Mater. Chem. C 2021, 9, 7292.

[64] I. Adell , R. M. Solé , M. C. Pujol , M. Lancry , N. Ollier , M. Aguiló , F. Díaz , Sci. Rep. 2018, 8, 11002. 30030467 10.1038/s41598-018-29372-z PMC6054691

[65] M.‐H. Yuan , H.‐H. Fan , H. Li , S. Lan , S.‐L. Tie , Z.‐M. Yang , Sci Rep 2016, 6, 21091. 26899189 10.1038/srep21091 PMC4761965

[66] Y. Sharma , B. Paudel , J. Lee , W. S. Choi , Z. Yang , H. Wang , Y. Du , K. T. Kang , G. Pilania , A. Chen , Appl. Phys. Lett. 2021, 119, 071902.

[67] A. Chen , Q. X. Jia , MRS Bull. 2021, 46, 115.

[68] A. Chen , Q. Su , H. Han , E. Enriquez , Q. X. Jia , Adv. Mater. 2019, 31, 1803241. 10.1002/adma.201803241 30368932

[69] P. Virtanen , R. Gommers , T. E. Oliphant , M. Haberland , T. Reddy , D. Cournapeau , E. Burovski , P. Peterson , W. Weckesser , J. Bright , S. J. van der Walt , M. Brett , J. Wilson , K. J. Millman , N. Mayorov , A. R. J. Nelson , E. Jones , R. Kern , E. Larson , C. J. Carey , İ. Polat , Y. Feng , E. W. Moore , J. VanderPlas , D. Laxalde , J. Perktold , R. Cimrman , I. Henriksen , E. A. Quintero , C. R. Harris , et al., Nat. Methods 2020, 17, 261. 32015543 10.1038/s41592-019-0686-2 PMC7056644

[70] P. Lu , E. Romero , S. Lee , J. L. MacManus‐Driscoll , Q. X. Jia , Microsc. Microanal. 2014, 20, 1782. 25307942 10.1017/S1431927614013245

