# Supplementary material for: Structural and Optical Properties of High Entropy (La,Lu,Y,Gd,Ce)AlO3 Perovskite Thin Films
Source: Adv Sci (Weinh). 2022 Aug 26;9(29):2202671. doi: 10.1002/advs.202202671 (PMC9561869; doi:10.1002/advs.202202671)
Supplement: Supplementary file 1 — Supporting Information [file ADVS-9-2202671-s001.pdf]

ADVANCED SCIENCE
Open Access

Supporting Information

for *Adv. Sci.*, DOI 10.1002/advs.202202671

Structural and Optical Properties of High Entropy (La,Lu,Y,Gd,Ce)AlO_3 Perovskite Thin Films

Zachary J. Corey, Ping Lu, Guangran Zhang, Yogesh Sharma, Bethany X. Rutherford, Samyak Dhole, Pinku Roy, Zhehui Wang, Yiquan Wu, Haiyan Wang, Aiping Chen and Quanxi Jia**

Supporting Information

Structural and optical properties of high entropy (La,Lu,Y,Gd,Ce)AlO_3 perovskite thin films

Zachary J. Corey, Ping Lu, Guangran Zhang, Yogesh Sharma, Bethany X. Rutherford, Samyak Dhole, Pinku Roy, Yiquan Wu, Haiyan Wang, Aiping Chen,[*] Quanxi Jia [*]

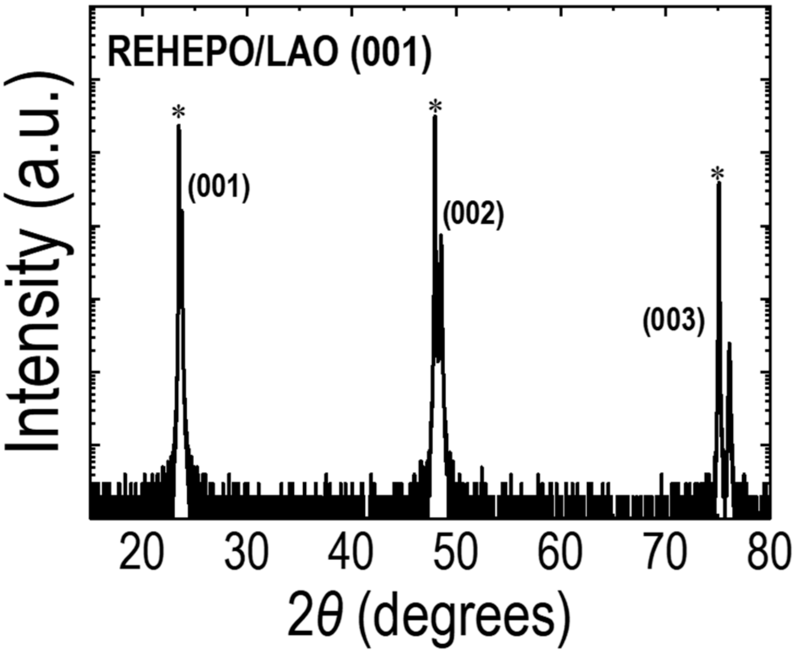

Figure S1. 2θ-ω scan of REHEPO thin film grown on LAO (001) substrate, where * indicates substrate peaks.

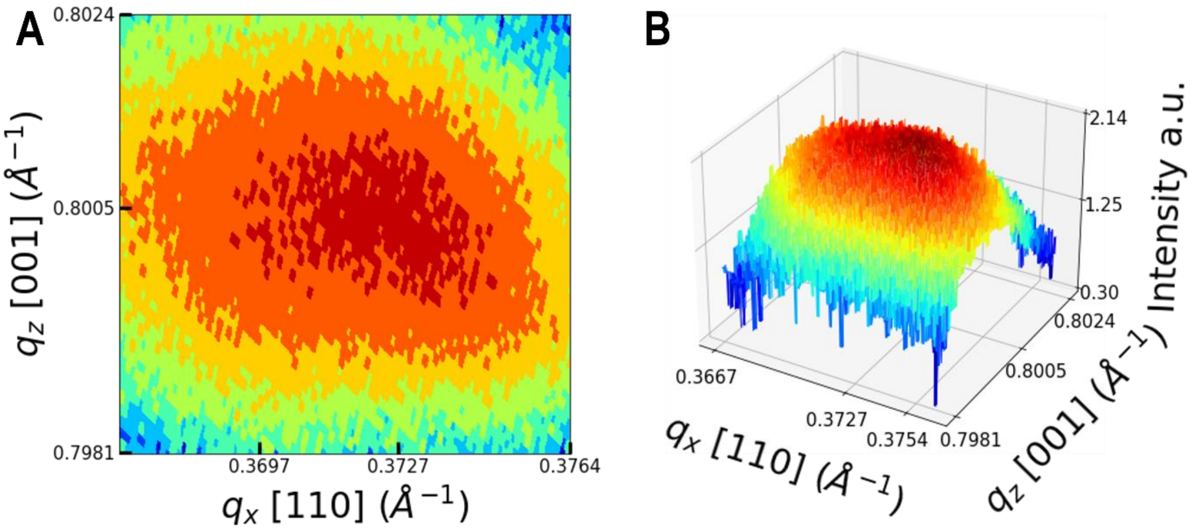

Figure S2. Local REHEPO film peak interpolated contour (a) and surface (b) calculated from the (113) RSM of the thin film grown on LAO substrate.

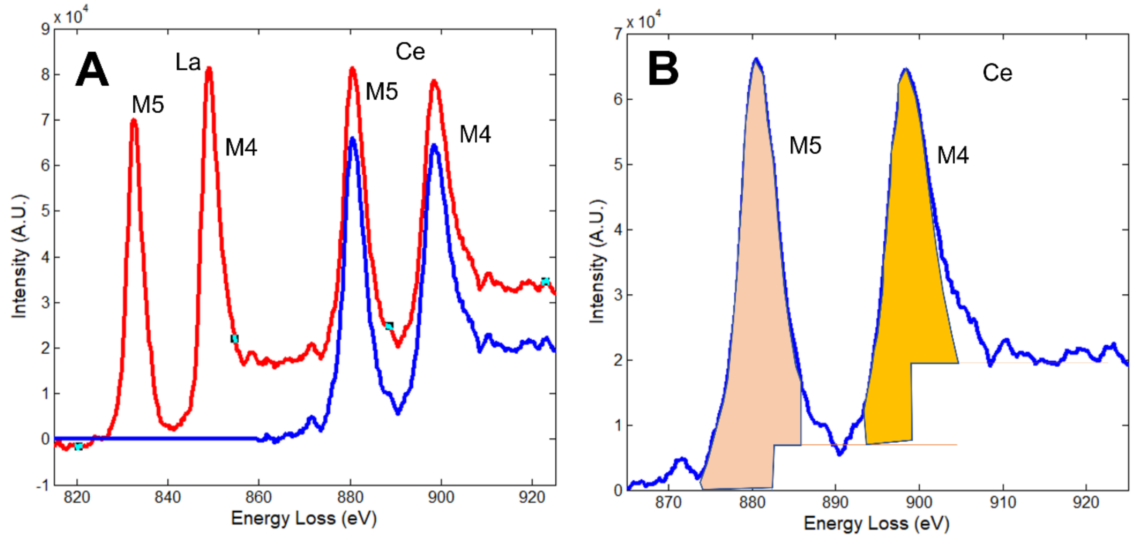

Figure S3. (a) EELS M lines (M5/M4) for La and Ce in the REHEPO thin films (red) as well as subtracted Ce M lines (blue). (b) The Ce subtracted M lines with the shaded integral area of M5 (beige) and M4 (orange) utilized for the area ratio calculation.

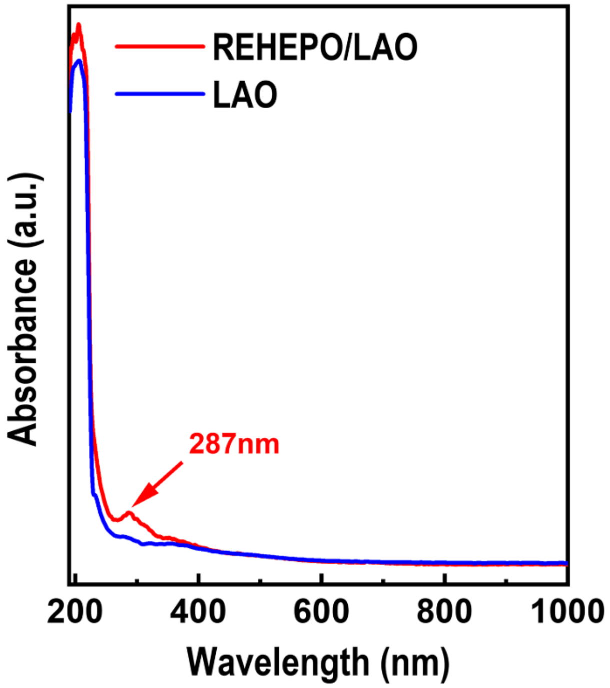

Figure S4. Absorbance of the REHEPO/LAO heterostrucutre compared to LAO substrate with the peak at 278 nm associated with Gd^{3+}.

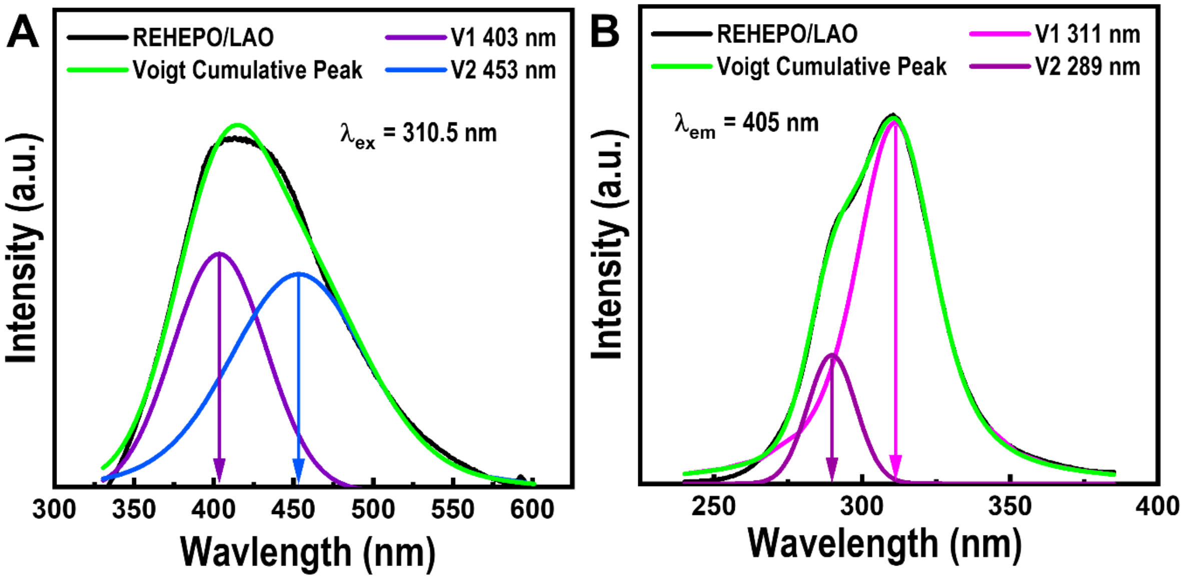

Figure S5. Deconvoluted Voigt fitting emission and excitation spectra for the REHEPO/LAO heterostructure. (a) Emission spectra from 310.5 nm excitation with two components of max intensity 403 nm and 453 nm. (b) Excitation spectra from 405 nm emission with two components of max intensity 311 nm and 289 nm.
